# Protocols and safety profile of convection enhanced delivery in pediatric patients: a systematic review

**DOI:** 10.1007/s00381-025-07121-8

**Published:** 2026-01-16

**Authors:** Nathan S. Fredricks, Mary Harkins, Anthony Price, Frances T. Morden, Michael C. Dewan

**Affiliations:** 1 John Sealy School of Medicine, The University of Texas Medical Branch at Galveston, Galveston, TX USA; 2 Department of Neurosurgery, The University of Texas Medical Branch at Galveston, Galveston, TX USA; 3 Department of Neurological Surgery, Vanderbilt University Medical Center, Nashville, TN USA

**Keywords:** Convection-enhanced delivery, Pediatrics, Drug delivery, Oncology

## Abstract

Convection-enhanced delivery (CED) is a developmental drug delivery system enabling direct, intraparenchymal placement of therapeutics that bypass the blood-brain barrier. This systematic review aggregates and outlines the available data surrounding current applications and adverse events of pediatric CED to benchmark safety as protocols expand. A comprehensive search through CINAHL, Cochrane, OVID Medline, and Scopus databases was conducted using a modified PRISMA analysis. After deduplication and screening, final articles were selected for data extraction. Of 108 unique articles, 12 studies met the inclusion criteria, including 162 patients (51% males, 44% females, and 5% not reported). The average age was 8.64 years (range 5–14.1 years). The most common indication for CED was for unresectable high-grade glioma (92%), predominantly diffuse intrinsic pontine glioma (83%). CED also addressed genetic enzyme deficiency (8%). The trans-frontal approach was most common (58%), with single catheter systems employed in 50% of studies. A total of 285 adverse events were reported. The most common included headache (20%), limb weakness (18.2%), Grade 1 cranial nerve deficits (6.3%), and general pain (4.9%). Of 132 CTCAE-graded events, 125 were Grade 1 or Grade 2 (94.7%) and 7 were Grade 3 (5.3%). No Grade 4 or Grade 5 adverse events occurred. This first systematic review of CED of pediatric patients demonstrates that while mild side effects are common, severe adverse events are rare. Multi-institutional and longitudinal data are needed to further define CED’s role in pediatric applications. These findings support CED as a viable platform for investigational therapies in treatment-resistant pediatric pathologies.

**Supplementary Information:**

The online version contains supplementary material available at 10.1007/s00381-025-07121-8.

## Introduction

Neurosurgeons have long sought different avenues for drug delivery for central nervous system (CNS) tumors and pathologies. Traditional drug-delivery systems struggled to convey therapeutics beyond the blood-brain barrier (BBB) in therapeutic doses that were not systemically toxic. Convection-enhanced delivery (CED) was designed to offer neurosurgeons an inventive approach to drug delivery. Instead of offering systemic doses of medications with noxious side effects and limited BBB penetrance, CED theoretically allows neurosurgeons to deliver therapeutic doses of drugs directly into the interstitial spaces of the brain. CED achieves this goal through microcatheters placed surgically into the treatment site and infusing the drug. This displaces extracellular fluid and aims to achieve a reproducible, homogenous, round-to-elliptical-shaped deployment of infusate. Drug placement can be confirmed with co-infusion of radiotracers to allow for real-time imaging of the infused bolus of fluid [1–3].

However, CED remains a developing treatment option, and the accuracy of drug delivery is critical [2, 3]. If placed incorrectly, the short distance to the flow of CSF may lead to the unintended spread of therapeutic agents. Furthermore, drugs that readily cross the blood-brain barrier should be restricted to minimize leakage and broader toxicities [1, 4]. At the same time, the understanding of its applications and safety profile in pediatric patients has not yet been systematically reviewed across all available data. Thus, we aimed to review the existing literature on pediatric applications of CED, the characteristics of treatment administration, and the adverse events recorded in the pediatric population. This study outlines these aspects of CED to enhance the understanding of its applications, common protocols, and safety among pediatric patients.

## Methods

### Study selection criteria

**Fig. 1:**
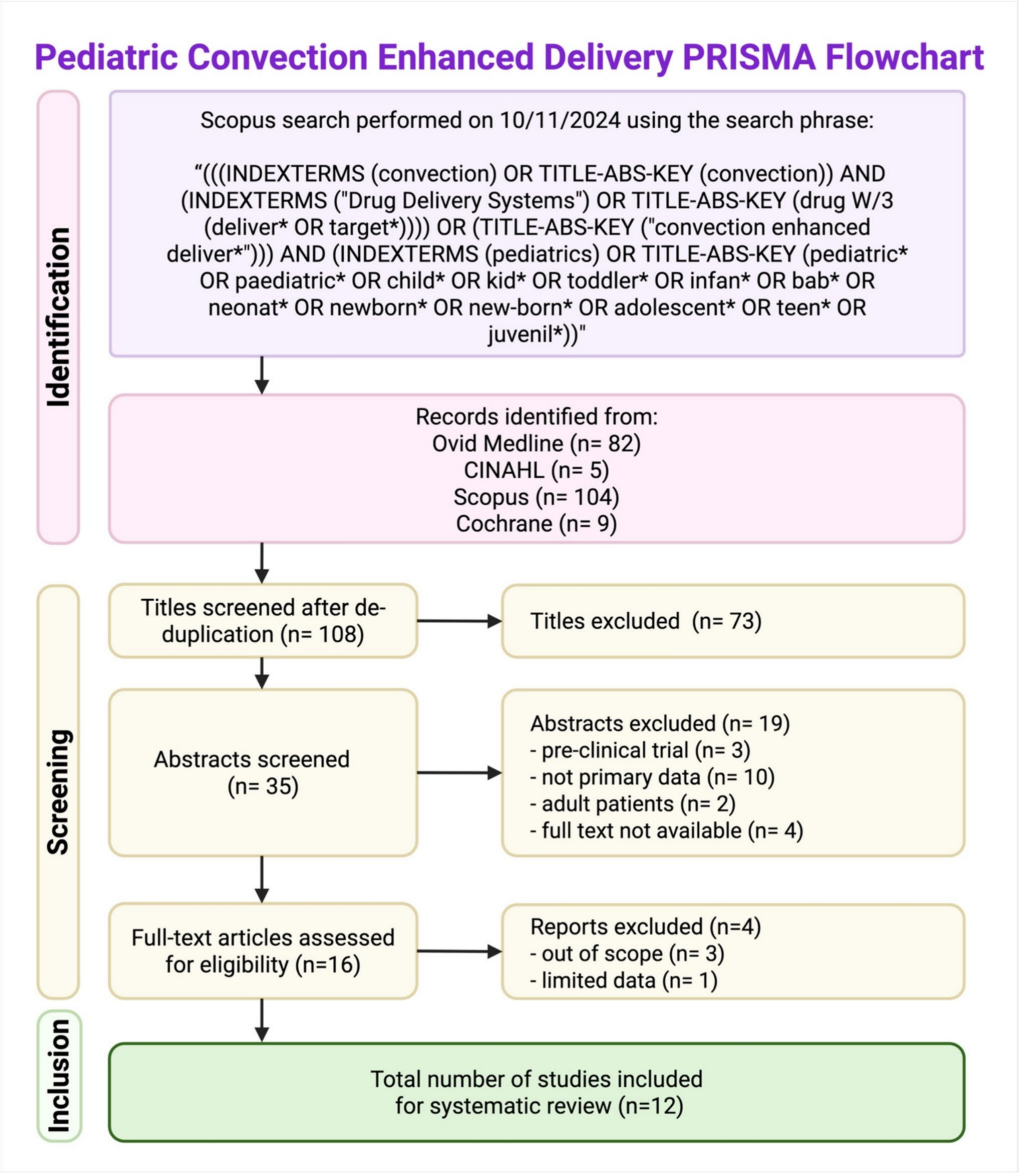
Pediatric convection-enhanced delivery PRISMA flowchart. [Created in BioRender. Fredricks, N. (2025) https://BioRender.com/u63k136]

A search of the Scopus, Cochrane, OVID Medline, and CINAHL databases for literature regarding convection-enhanced delivery (CED) in pediatric patients was conducted on October 11, 2024. Search criteria were crafted with assistance from a medical reference librarian and adjusted for database-specific criteria. An example of search terms can be found in Fig. 1.

Screening was completed by two reviewers (N.F. and M.H.), and discrepancies were resolved via discussion. Preliminary screens by title and abstract were conducted, followed by a deeper, full-text review of the remaining articles. Inclusion criteria included pediatric patient populations (age ≤ 18 years), use of convection-enhanced delivery for treatment of CNS disorders, adequate primary data, and data relevant to safety outcomes and/or pharmacokinetic data. Given the relative novelty of CED, all study designs yielding primary data were included. Both single and multi-infusion studies were included, as well as those that modulated infusion volume. Because CED is used for the treatment of multiple types of nervous system pathologies, the goal of this review was to evaluate the safety and efficacy of the CED procedure rather than the specific mechanisms and clinical outcomes of a given infused drug. All applications were included in this review. Exclusion criteria included studies involving adult patients, review articles, preclinical or animal studies, non-English publications, and results mistakenly retrieved by our search criteria that were outside the scope of CED research (e.g., non-CED drug delivery studies).

### Data evaluation and collection

Across all papers, two reviewers (N.F. and M.H.) assessed the full texts, with discrepancies resolved via discussion. Risk of bias assessment, standard effect measures, and formal certainty assessment were not conducted as a part of this modified PRISMA review and synthesis of treatment characteristics and adverse events. Criteria analyzed included study design (retrospective vs. prospective), number of patients, average patient age, patient sex, multiple versus single infusions, total number of CED infusions, rationale for treatment, therapeutic infusion, tracer used, catheter destination, surgical approach, number of catheters per system, peak flow rate, total infusion volume, and infusion time. The total number of CED infusions was defined as the sum of CED surgical procedures and subsequent catheterization (e.g., studies with repeat infusions), or the number of times infusion was initiated in the case of the re-usable microcatheters of the Renishaw Drug Delivery System (RDDS). The number of catheters per CED system was defined as the total number of catheters implanted surgically per patient per infusion (e.g., bilateral catheters flanking tumor).

To evaluate safety, all adverse events related to the CED procedure were analyzed across all papers as reported by each study. Adverse events were defined as any complications or sequelae, neurologic or systemic, occurring as a consequence of CED surgical intervention. These events were recorded as reported in each paper and further organized into broader categories for comparison across multiple studies. We included all events described as definitively or likely attributed to CED intervention by the original study authors.

**Fig. 2:**
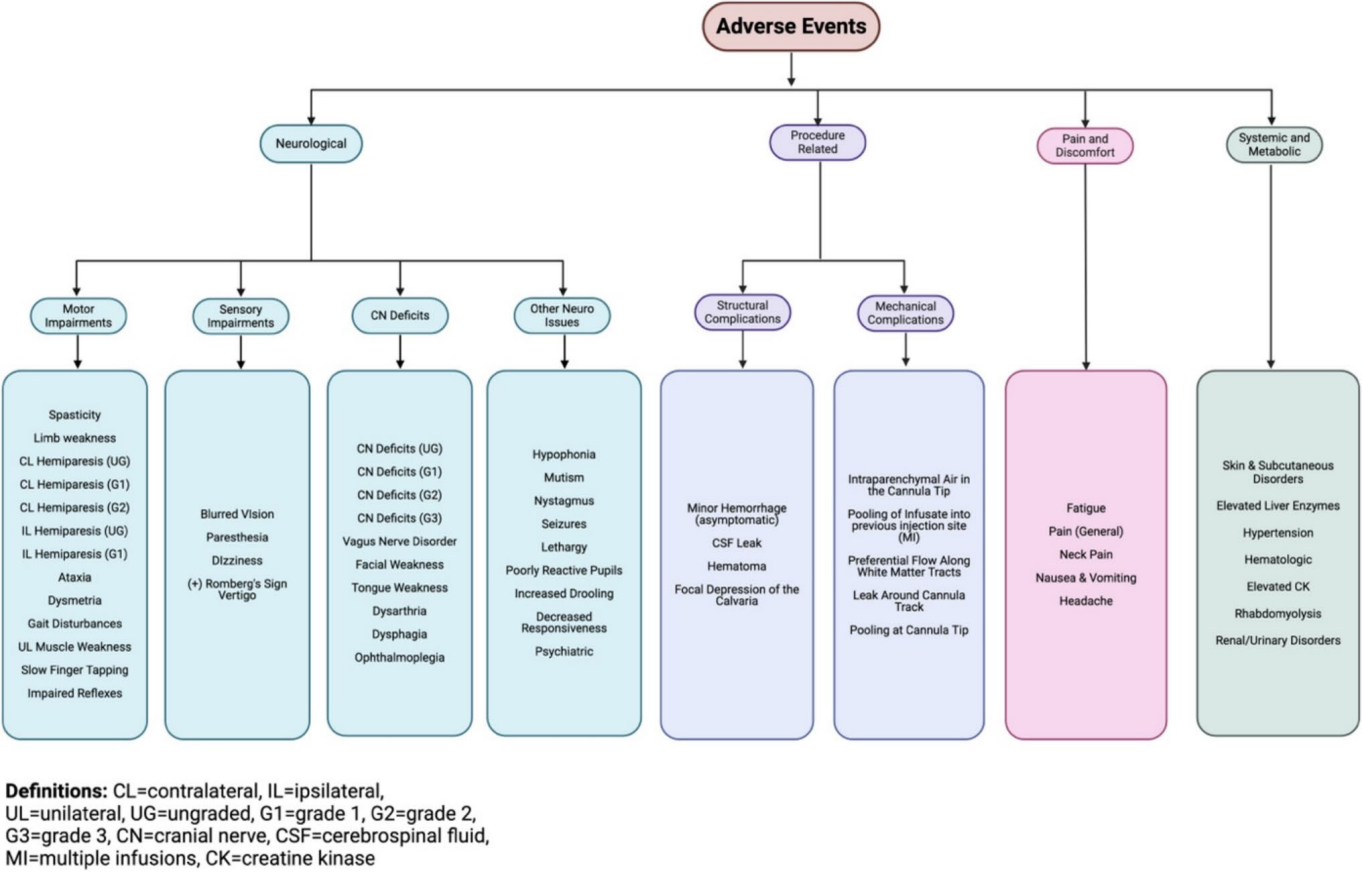
CED-related adverse event categorization flow chart [Created in BioRender. Harkins, M. (2025) https://BioRender.com/h89h849]

Adverse events were classified into four main categories: Neurological Symptoms, Procedure-Related Complications, Pain and Discomfort, and Systemic and Metabolic Complications. Each category and the specific adverse events included are outlined in Fig. 2.

Scoring according to the Common Terminology Criteria for Adverse Events (CTCAE) grading scale was reported across studies when described. The CTCAE scale ranks each adverse event from Grade 1 to Grade 5 with a classification of grading for given adverse events. For example, hydrocephalus descriptions by grade include asymptomatic (Grade 1); moderate symptoms, intervention not indicated (Grade 2); severe symptoms or neurological deficit, intervention indicated (Grade 3); life-threatening consequences, urgent intervention indicated (Grade 4); and death (Grade 5) [11]. All adverse events were classified into three categories based on grading: CTCAE Grade 1 or 2 adverse events, CTCAE Grade 3 adverse events, and ungraded adverse events. Graded and ungraded events were evaluated as separate data sets to afford analysis of common clinical manifestations across various studies, as well as to assess the occurrence of serious events.

In addition to adverse events, the time course of symptom resolution and Karnofsky Performance Status Scores (KPS) was recorded. Symptom duration under 30 days was considered transient, while longer symptom courses were considered permanent. KPS scores before and after CED were gathered to further evaluate the impact of CED on overall function and quality of life.

Given the broad nature of clinical applications of CED in the studies analyzed in this review, metrics to evaluate efficacy were not primarily focused on clinical outcomes. Instead, two metrics intrinsic to the surgical procedure itself were selected that evaluated the pharmacokinetic efficacy of CED over systemic infusion and the overall feasibility of catheterization and local infusion. These were the Vd:Vi ratio and the reported targeting accuracy of the catheters. The Vd:Vi ratio, defined as the volume of distribution (Vd) of infusate tracer (e.g., gadoteridol) normalized to the volume of infusion (Vi), reflects the spatial distribution of CED-infusate. Together, these are important metrics for determining the safety and efficacy of CED and were reported with available data.

## Results

### Study selection criteria

The preliminary CINAHL, Cochrane, OVID Medline, and Scopus database search yielded 200 papers ranging in publication date from 1999 to 2024, 73 of which were eliminated by title screening. The remaining 35 were screened by abstract, with 15 excluded for improper study design and 4 for unavailability. The resultant 16 full texts were evaluated, and 4 studies were excluded, 3 of which were out of scope, and 1 of which provided limited data for analysis. The final 12 studies included in the review met the inclusion criteria and ranged in publication date from 2013 to 2024 [5–10, 12–17]. The sequence of screening utilized in this review process is outlined in Fig. 1.

### Study demographics and data collection

Of the 12 studies, 8 were retrospective series, and 4 were prospective cohort studies. The study size ranged from 2 to 39 patients, with a total of 162 patients included in the final review, including 83 males (51%), 71 females (44%), and 8 (5%) not reported (Table 1).

**Table 1:** Papers that met inclusion criteria with study information and patient demographic data

List of studies that met the inclusion criteria (*n* = 12)								
**Authors & year**	Publication	Location	Study	No. Pts	Age (mean)	Male	Female	Sex not reported
Anderson et al. (2013)	*JNS: Pediatrics*	Morgan Stanley Children’s Hospital and Columbia University Medical Center	Case Series	2	5	-	2	-
Bander et al. (2020)	*JNS: Pediatrics*	Memorial Sloan Kettering Cancer Center	Retrospective	7	5.4	4	3	-
Bander et al. (2020)	*Neurosurgical Focus*	Memorial Sloan Kettering Cancer Center	Retrospective	23	7.7	10	13	-
Barkley et al. (2024)	*JNS: Pediatrics*	Duke University and the University of Alabama at Birmingham	Retrospective	19	14.1	10	9	-
Chittiboina et al. (2014)	*JNS: Pediatrics*	National Institute of Neurological Disorders and Stroke, NIH	Case Series	4	13	2	2	-
Heiss et al. (2019)	*JNS: Pediatrics*	National Institute of Neurological Disorders and Stroke, NIH	Case Series	5	13	3	2	-
Hollingworth and Zacharoulis (2022)	*Journal of Neuro-Oncology*	Multi-Institution	Retrospective	8	7.2	-	-	8
Morgenstern et al. (2019)	*JNS*	Multi-Institution	Prospective	39	7.6	23	16	-
Mueller et al. (2023)	*Journal of Neuro-Oncology*	University of California, San Francisco	Prospective	7	9.3	6	1	-
Pearson et al. (2021)	*Nature Communications*	Multi-Institution	Prospective	7	6.5	3	4	-
Souweidane et al. (2018)	*The Lancet Oncology*	Memorial Sloan Kettering Cancer Center	Prospective	28*	8	17	11	-
Szychot (2021)	*International Journal of Clinical Oncology*	Harley Street Children’s Hospital in London, United Kingdom	Retrospective	13	6.9	5	8	-
**Total:**				**162**	**8.6**	**83**	**71**	**8**

*JNS Journal of Neurosurgery*, *Pts* patients

^*^25 included in per-protocol analysis, 28 included in safety analysis

The average patient age was 8.6 years old (range 5–14.1 years). The number of studies giving a single infusion (*n* = 6, 50%) matched the number giving more than one infusion (*n* = 6, 50%). The total number of infusions ranged from 1 to 12. The weighted average total infusion volume was 4.90 mL (range 0.16–7.97 mL). The average peak flow rate was 0.39 mL/h (range 0.01–0.9 mL/h) (Table 2). 

**Table 2:** Papers that met inclusion criteria with study design and CED parameters

Authors & year	Infusions per patient	Total infusions	Surgical approach	Catheters per patient	Rationale for treatment	Infusate	Catheter location		Avg. peak flow rate (mL/h)	Avg. total infusion Vol. (mL)
Anderson et al. (2013)	1	2	Transcerebellar	2	DIPG	Topotecan	Brainstem		0.07	5.67
Bander et al. (2020)	2–3	15	Supratentorial	1	DIPG	[^124^I]−8H9	Brainstem		-	3.24
Bander et al. (2020)	1	23	Supratentorial	1	DIPG	[^124^I]−8H9	Brainstem		0.01	3.9
Barkley et al. (2024)	1	19	Varied	1–4	Pediatric Non-Pontine High-Grade Glioma	Lerapolturev and G207	Corpus callosum, thalamus, insula, cingulate gyrus		0.4	2.4
Chittiboina et al. (2014)	5	5	Supratentorial	1	DIPG	IL13-PE38QQR	Brainstem		0.48	2.58
Heiss et al. (2019)	2–3	7	Supratentorial	1	DIPG	IL13-PE38QQR	Brainstem		0.43	2.47
Hollingworth and Zacharoulis (2022)	1–12	55	Transcerebellar (2) Transfrontal (2)	4	DIPG	Carboplatin and Sodium Valproate	Brainstem		0.18	-
Morgenstern et al. (2019)	1	39	Supratentorial	1	DIPG	[^124^I]−8H9	Brainstem		-	4.19
Mueller et al. (2023)	1–8	48	Transcerebellar or transfrontal^b^	2	DIPG	MTX110	Brainstem		0.9	5.81
Pearson et al. (2021)	1	7	Transfrontal	4	AADC deficiency	AAV2-hAADC	Brainstem		-	0.16
Souweidane et al. (2018)	1	25^a^	Transfrontal	1	DIPG	[^124^I]−8H9	Brainstem		0.45	1.99
Szychot (2021)	7	69	Transcerebellar (2) Transfrontal (2)	4	DIPG	Carboplatin and Sodium Valproate	Brainstem		0.3	7.97
Total:		**314**						**Avg.:**	**0.39**	**4.9**

^a^25 included in per-protocol analysis, 28 included in safety analysis

^b^Not specified for each case. *Avg.* average, *Vol.* volume, *DIPG* diffuse intrinsic pontine glioma, *[*^*124*^*I]−8H9* radioimmunotherapy omburtamab, *IL13-PE38QQR* interleukin-13–pseudomonas exotoxin, *MTX110* aqueous panobinostat, *AADC* aromatic L-amino acid decarboxylase deficiency, *AAV2-hAADC* viral vector expressing AADC

The vast majority of CED implementation in pediatric patient populations was within the context of treating unresectable high-grade primary brain tumors (*n* = 11 studies: diffuse intrinsic pontine glioma, *n* = 10 studies; and non-pontine high-grade glioma, *n* = 1 study). The exception was Pearson et al.’s (2021) study on the use of CED in the treatment of aromatic L-amino acid decarboxylase deficiency [16]. Nearly all studies involved the stereotactic placement of new catheters for each infusion. However, one study (Szychot) implemented the Renishaw Drug Delivery System (RDDS), a novel CED system developed to facilitate intermittent CED via the surgical introduction of four indwelling catheters via a skull-mounted transcutaneous port [17]. The therapeutic infusates consisted of topotecan (1 study), lerapolturev (1), MTX110 (1), viral vector expressing AADC (AAV2-hAADC) (1), carboplatin and sodium valproate (2), interleukin-13–Pseudomonas exotoxin (IL13-PE) (2), and ^124^I-8H9 (Omburtamab) (4). The tracers used were gadolinium-diethylenetriamine pentaacetic acid (Gd-DTPA) (4) and gadoteridol (2). One study did not use a tracer, and five studies did not specify whether a tracer was used.

### Surgical approach and strategy

The catheter destination matched the pathology and included the pons in 10/12 studies (83%). The remaining 2/12 studies (17%) included multiple catheter destinations: Barkley et al. targeted the frontal, parietal, temporal, or occipital lobes, as well as the cingulate gyrus, insula, corpus callosum, and thalamus, while Pearson et al. targeted the substantia nigra pars compacta (SNc) and the ventral tegmental area (VTA) [13, 16]. The surgical approach (catheter trajectory) as well as the number of catheters per CED system varied based on relevant anatomy at the destination site, as well as the size of the lesion dictating coverage.

All 10 studies targeting the pons were for the treatment of DIPG [5–10, 12, 14, 15, 17]. Among these, 6/10 implemented a single-catheter system [5–8, 10, 14], 2/10 implemented a 2-catheter system [9, 12], and the remaining 2/10 implemented a 4-catheter system [15, 17]. Hollingworth and Zacharoulis reported increased and more uniform tumor coverage afforded by a multi-catheter system [15]. Similarly, Heiss et al. concluded that a single catheter could infuse the local tumor, but not the entire MRI-visible tumor, and a multi-catheter approach could have distributed infusate to more tumor tissue [7]. Szychot noted the flexibility of a multi-catheter system by the ability to switch between catheters during infusion in the event of neurological defects associated with infusion along a specific catheter, thereby continuing the infusion without additional deficits [17]. With regard to surgical approach, 6/10 took a purely trans-frontal (supratentorial) approach, traversing either the anterior or posterior tegmentum and terminating in the pontomedullary junction [5–8, 10, 14]. One study described a purely trans-cerebellar (infratentorial) approach, traversing the cerebellar hemispheres following the middle cerebellar peduncles, two studies described both trans-frontal and trans-cerebellar approaches, and a final study utilized either a trans-frontal or trans-cerebellar approach depending on the patient [9, 15, 17]. Preferential distribution of infusate along parallel axially oriented white matter tracts was observed in Chittiboina et al., but ultimately filled along perpendicularly oriented corticospinal tracts with continued Vi [14]. Morgenstern et al. reported that transgression of corticospinal fibers due to catheter placement or infusate distribution could not be correlated with long-term deficits (> 30 days), though one patient (3%) experienced a permanent deficit (facial nerve palsy) [8].

The remaining 10/12 studies included multiple destination sites. Barkley et al. investigated the use of CED in various non-pontine high-grade pediatric gliomas whose trajectory and surgical approaches (supra-tentorial or infra-tentorial) varied based on tumor location [13]. Similarly, the number of catheters per CED system varied from 1 to 4 catheters to optimize tumor coverage. Pearson et al. used a trans-frontal approach for all catheter insertions, and a 4-catheter system was employed: bilateral catheters at both locations [16]. Overall, the most common surgical approach was transfrontal (*n* = 7/12 studies, 58%) and the most common CED system used a single catheter (*n* = 6/12 studies, 50%).

Seven studies recorded their Vd:Vi ratio (*n* = 176 infusions) [5, 7, 9, 10, 14, 16, 17]. The average Vd:Vi ratio was 2.65 (range 1.8–3.4). Two studies compared Vd:Vi ratios across varying Gd-DTPA concentrations and found that Vd:Vi ratios were significantly reduced at lower Gd-DTPA concentrations (mean Vd:Vi for 1 mM Gd-DTPA = 1.6, and 3.05 for 5 mM Gd-DTPA) [7, 14]. Additionally, six studies included targeting accuracy as a metric for CED efficacy [5, 9, 10, 12, 13, 16]. Five studies utilized MR imaging to assess the stereoscopic placement of catheters in the brainstem, and none needed repositioning [5, 9, 12, 16]. Souweidane et al. assessed catheter placement by monitoring [^124^I]−8H9 localization on PET scans and found that all infusate localized to the brainstem lesion immediately [10]. Barkley et al. utilized CT to assess catheter placement in the corpus callosum, thalamus, insula, and cingulate gyrus, and was the only study to report catheter misplacement [13]. In this study, 3/49 catheters were misplaced. Two of these were pulled back and successfully repositioned after CT revealed they were < 1 cm from CSF space, and 1 catheter was not used as it was too close to the ventricle and not feasible to pull back. Overall, 48/49 catheters were usable in Barkley et al.’s study, and there were no infections or neurological sequelae related to either catheter placement or removal [13]. Therefore, among 6 studies describing the placement of 217 catheters, only a single catheter (0.46%) was unusable.

### Adverse events and safety metrics

Among the 162 patients (314 total infusions), a total of 285 adverse events were recorded. A complete listing of every recorded event and classification may be found in Supplemental Data Table 1. Between the four categories of adverse events, the most common were neurological symptoms with 166 adverse events recorded (58.2%), followed by pain and discomfort-related events at 78 occurrences (27.4%), 22 (7.7%) procedure-related complications, and 19 (6.7%) systemic and metabolic complications. Across all categories, the most common adverse events were headache (*n* = 57, 20%), limb weakness (*n* = 52, 18.2%), Grade 1 cranial nerve deficits (*n* = 18, 6.3%), general pain (*n* = 14, 4.9%), and ataxia (*n* = 12, 4.2%). Of the 285 adverse events recorded, 132 were CTCAE-graded (46.3%) [5–10] and 153 were ungraded (53.7%). Of the total graded events, 125 (94.7%) were CTCAE Grade 1 or 2, and 7 were CTCAE Grade 3 (5.3%). There were no Grade 4 or Grade 5 adverse events recorded (Table 3).

**Table 3:** Categorization of CTCAE graded adverse events

CTCAE grade	Occurrences (*n* = 132)	Frequency (%)
Grade 1	60	45.5
Grade 1 or 2*	30	10.5
Grade 2	35	26.5
Grade 3	7	5.3
Grade 4	0	-
Grade 5	0	-

^*^Mueller et al. Grouped Grade 1 and Grade 2 adverse events together as “CTCAE Grade 1 or 2”

*CTCAE* Common Terminology Criteria for Adverse Events

Three studies, however, failed to fully distinguish adverse events resulting from the procedure or the therapeutic infusion itself (*n* = 87 adverse events). Anderson et al. (*n* = 4 adverse events) presented symptoms and events resulting after each infusion but did not specifically attribute them to the surgical intervention or topotecan infusate [12]. Heiss et al. presented both a chart of occurrences of Grade 1 and Grade 2 events due to CED procedure, as well as a narrative recollection of adverse events in a case series review, but did not specify whether the events described in the case series coincided with the numbered instances of graded events (*n* = 25 adverse events) [7]. Souweidane et al. included both the adverse events and CTCAE Grades, but stated that the graded events could not be solely attributed to the procedure and may be due to the infused [^124^I]−8H9 immunotherapy (*n* = 58 adverse events) [10].

The time course of symptoms was recorded for 115 of the 285 adverse events [7–10, 15–17]. This was deemed significant in determining the nature of the adverse events experienced during and after the CED surgical procedure. Among 115, 9 persisted 30 days post-op (7.83%).

Four of the 12 studies included Karnofsky/Lansky performance scores (*n* = 48 patients) [7, 10, 12, 17]. Of those 48 patients, 33 remained at the same KPS score at baseline (68.8%), 12 had lower scores compared to baseline (25%), and 3 saw improved scores compared to baseline (6.2%). Of the 12 that saw a decrease in scores, 75% saw a decrease of ≤ 10 points (*n* = 9), 83.3% a decrease of ≤ 20 points (*n* = 10), 91.7% saw a decrease of ≤ 30 points (*n* = 11), and 100% saw a decrease of ≤ 40 points (*n* = 12).

The infrequency and inconsistency of global outcomes reporting—including overall survival (OS) and progression-free survival—across studies precluded meaningful aggregation and analysis. Nonetheless, median overall survival for studies including patients with DIPG ranged from 10.0 to 26.1 months, with most falling below 16 months median OS [7, 9, 10, 17].

## Discussion

To our knowledge, this is the first review to aggregate and examine the uses and adverse event profile of CED in pediatric applications. To this end, this study has demonstrated important outcomes regarding the adverse events related to this surgical, drug-delivery technology. As CED develops and future studies are planned, it is important to benchmark the accuracy and common adverse events related to this procedure.

### Targeting accuracy

Due to the sensitive nature of placing intraparenchymal cannulas and therapeutic agents within eloquent structures, accuracy is critical. Replacing cannulas can create adjacent tracts for infusate leak back, thus disrupting the uniform placement of the desired bolus and increasing the number of cannula tracts placed in the pediatric brain [9]. Of the included studies, 6 of the 12 studies included comments on the targeting accuracy for their cannula placement. For infusate and cannula localization, four studies used MRI, one used a PET scan, and one used a CT scan.

Of the available data, 217 catheters were placed across 116 total infusions. Of these 217, 3 were reported as misplaced (1.38%, *n* = 3/217). Further, two of these misplaced cannulas were successfully repositioned, leaving 1 of 217 cannulas unusable (0.46%, *n* = 1/217). The unusable cannula was unable to be removed and replaced due to its proximity to the child’s ventricle, highlighting the importance of utmost care in accurate placement.

The accuracy described here matches that found in the broader literature [3]. The future of CED targeting ought to seek out further improvements in surgical accuracy and efficiency. Predictive infusion algorithms and robotic placement systems are currently under study to further develop the surgical accuracy and efficiency of this developing procedure [18–20].

### Adverse events

Differentiating adverse events triggered by the use of CED versus events caused by infusate toxicity presented a challenge for this report [2]. As described herein, most pediatric applications for CED are for the delivery of chemotherapeutic agents in highly malignant glioma. The administration of chemotherapeutic drugs can cause varied responses, which may be independent of CED administration and, therefore, skew adverse effect findings around the use of CED. To most accurately report adverse events, we aggregated all side effects except those identified as medication-related by study authors. This allowed for the most complete reporting of adverse events in the current pediatric CED literature.

To summarize the aggregated AE data, preliminary studies indicate that pediatric CED for unresectable malignant glioma is relatively safe. While common, > 95% of side effects are low-grade and transient, and no major, permanent neurologic deficits have been attributable to the CED procedure. According to CTCAE scoring, this indicates that the majority of patients required no or only local, noninvasive side effect-related intervention. With all graded adverse events included, no child faced life-threatening effects from CED or needed urgent intervention. Importantly, however, in the analysis of patients with KPS scores reported, 25% saw a decline in functional status after CED. This feature warrants attention in ongoing and future prospective investigations, ideally designed to distinguish performance deterioration related to CED from natural disease progression.

Including the individually reported and aggregated side effects, the most common were headache (*n* = 57, 20%), limb weakness (*n* = 52, 18.2%), mild cranial nerve deficits not needing intervention (*n* = 18, 6.3%), pain (*n* = 14, 4.9%), and ataxia (*n* = 12, 4.2%). Throughout the aggregated data, these pediatric findings are consistent with those from the broader population [2].

It is also important to distinguish whether the effects induced by CED are transient or long-lasting, particularly for a disease wherein weeks and months may represent a significant portion of a child’s remaining survival duration. In the broader literature, cases of continued neurological deficits, while rare, have been noted [21–23]. The time course of symptoms was recorded for 115 of the 285 adverse events, and permanent deficits were regarded as those that persisted 30 days post-CED infusion. Of those 115, 9 persisted at 30 days post-op (7.83%). Of the studies that did not report patient-level data, reports about long-term safety supported a transient time course of adverse effects. This note was carried across a range of total infusate volumes (< 1–8.8 mL/single infusion).

These findings of common side effects and time course suggest two important takeaways: most CED patients experience mild and transient symptoms, and both severe or permanent adverse events are uncommon. However, while limited, reports of declining KPS scores indicate the need for caution. This balance demonstrates the importance of careful application of CED. In the setting of pathologies with dismal prognoses, like DIPG and L-AADC deficiency, there is an urgent need for novel treatment regimens. While still poor, the relatively small CED sample size represented by this systematic review demonstrates a slightly more favorable OS than what the alternate literature describes (8–15 months) [24–26]. Larger, prospective studies powered to detect true survival differences will help determine the role of CED for unresectable high-grade glioma in children. Thus, the relatively favorable safety profile demonstrated in this review suggests that CED is an appropriate vehicle to explore novel drugs to target malignancies that have otherwise proven refractory to conventional therapy.

### Future directions

As future trials are developed, it is important to consider what features may best suit the collaborative development of CED therapies. By crafting clinical trials with consistent features, future surgeons may more easily and accurately compare results and measure therapeutic benefits and harms. Standardization of pathology-specific practices may include optimizing for single vs. multiple-catheter use, approach selection, and infused tracers. For example, Szychot et al. demonstrate optimal imaging regimens for infusate monitoring in future CED for DIPG trials [27]. Additionally, future reporting structures that routinely separate infusate effects from procedure impacts will be an important step in maximizing CED’s safety profile. Improving these procedures and metrics would allow for comparison across studies and future meta-analysis.

Additionally, the development of CED technology continues to expand and hopefully improve patient outcomes. For example, the development of the multi-catheter, implantable systems can further reduce the likelihood of CED-related adverse effects. Unlike traditional CED approaches requiring new catheter placement for each infusion, implanted systems utilize multiple indwelling catheters accessible via a skull-mounted transcutaneous port, enabling intermittent infusions without repeated craniotomies [17, 28]. This innovation addresses two critical challenges: first, it eliminates the cumulative risk of multiple surgical procedures for patients requiring serial infusions; second, it provides flexibility to switch between catheters if neurological deficits emerge during infusion along a specific tract. The ability to deliver multiple treatments through a single surgical implantation may prove particularly valuable in pediatric populations with treatment-resistant pathologies requiring prolonged or repeated therapeutic courses.

### Limitations

This study is limited due to the nature of its structure as a retrospective systematic review. However, careful review and a systematic approach equip this study to accurately represent the findings and data currently available in the pediatric CED literature. Formal risk of bias and certainty assessments were not performed because the included studies were predominantly single-arm feasibility or retrospective series with heterogeneous patient populations and no comparator groups. For these reasons, conducting a formal risk of bias and certainty assessment was not appropriate. However, risk of bias was discussed narratively throughout. This included risk of bias from variable adverse event reporting and heterogeneity in treatment paradigms. For example, not all studies graded adverse events according to the CTCAE criteria, and certain treatment agents may be more tolerable and effective than others. Although this limited critical appraisal of the methodological quality of the included studies, this review still offers the most comprehensive summary of pediatric CED-related adverse events to date. By including broad study methodologies, this review was able to include more patient adverse event data and better describe the spectrum of safety considerations in CED writ large. Standard effect measures were not calculated, as this review focused on descriptive characterization of adverse events rather than comparative treatment effectiveness. Additionally, this review sought to be broadly inclusive of all side effects reported unless specifically labeled as medication-related by the primary study. While this potentially blends procedure and drug-related events, our methods aimed to avoid under-reporting adverse events. Continued work to identify and distinguish these effects will serve to improve future CED investigations.

## Conclusion

This review is the first to aggregate the adverse effects of convection-enhanced delivery (CED) in pediatric patients, describing its applications and adverse events profile. CED is predominantly used for unresectable, high-grade glioma where treatment options are limited, and prognoses are poor. Adverse event reporting indicates that most patients undergoing the procedure experience mild, transient neurological symptoms, and permanent or serious effects are rare. Cannula placement was generally accurate, with few instances requiring repositioning. As neurosurgeons refine this technique, the current safety data should serve as a benchmark. The findings here support CED as a viable platform for delivering investigational therapies in treatment-resistant pediatric pathologies, with future improvements aimed at further minimizing procedural risk.

## Supplementary Information

Below is the link to the electronic supplementary material.

ESM 1 DOCX (19.7 KB)

## Data Availability

No datasets were generated or analysed during the current study.
