# Supplementary material for: Protocols and safety profile of convection enhanced delivery in pediatric patients: a systematic review
Source: Childs Nerv Syst. 2026 Jan 16;42(1):30. doi: 10.1007/s00381-025-07121-8 (PMC12808288; doi:10.1007/s00381-025-07121-8)
Supplement: Supplementary file 1 — DOCX (19.7 KB) [file 381_2025_7121_MOESM1_ESM.docx]

**Supplemental Data Table 1:** Complete Adverse Event Category Classification and Occurrences

| **Adverse Events** | **Instances (n=285)** | **Frequency (%)** |
| --- | --- | --- |
| **Neurological** |  |  |
| *Motor Impairments* | **97** | **34** |
| Limb Weakness | 52 | 18.2 |
| Ataxia | 12 | 4.2 |
| CL Hemiparesis (Grade 1) | 5 | 1.8 |
| CL Hemiparesis (Grade 2) | 8 | 2.8 |
| CL Hemiparesis (Ungraded) | 1 | 0.4 |
| IL Hemiparesis (Grade 1) | 2 | 0.7 |
| IL Hemiparesis (Ungraded) | 4 | 1.4 |
| Gait Disturbance | 4 | 1.4 |
| Unilateral Muscle Weakness | 4 | 1.4 |
| Spasticity | 2 | 0.7 |
| Dysmetria | 1 | 0.4 |
| Slow Finger Tapping | 1 | 0.4 |
| Impaired Reflexes | 1 | 0.4 |
| *Sensory Impairments* | **5** | **1.8** |
| Blurred Vision | 1 | 0.4 |
| Dizziness | 1 | 0.4 |
| Paresthesia | 1 | 0.4 |
| Positive Romberg’s Sign | 1 | 0.4 |
| Vertigo | 1 | 0.4 |
| *Cranial Nerve Deficits* | **52** | **18.2** |
| CN Deficits (Grade 1) | 18 | 6.3 |
| CN Deficits (Grade 2) | 8 | 2.8 |
| CN Deficits (Grade 3) | 1 | 0.4 |
| CN Deficits (Ungraded) | 3 | 1.1 |
| Dysarthria | 6 | 2.1 |
| Facial Weakness | 6 | 2.1 |
| Ophthalmoplegia | 6 | 2.1 |
| Dysphagia | 2 | 0.7 |
| Tongue Weakness | 1 | 0.4 |
| Vagus Nerve Disorder | 1 | 0.4 |
| *Other Neurological Issues* | **12** | **4.2** |
| Nystagmus | 3 | 1.1 |
| Psychiatric Issues | 2 | 0.7 |
| Decreased Responsiveness | 1 | 0.4 |
| Hypophonia | 1 | 0.4 |
| Increased Drooling | 1 | 0.4 |
| Lethargy | 1 | 0.4 |
| Mutism | 1 | 0.4 |
| Poorly Reactive Pupils | 1 | 0.4 |
| Seizure | 1 | 0.4 |
| **Procedure-Related Complications** | **22** | **7.7** |
| *Mechanical Complications* | **10** | **3.5** |
| Minor Hemorrhage (Asymptomatic) | 7 | 2.5 |
| CSF Leak | 1 | 0.4 |
| Focal Depression of Calvaria | 1 | 0.4 |
| Hematoma | 1 | 0.4 |
| *Structural Complications* | **12** | **4.2** |
| Infusate leakage into previous catheter track (multiple infusions) | 4 | 1.4 |
| Preferential Flow Along White Matter Tracts | 3 | 1.1 |
| Intraparenchymal Air at the Cannula Tip | 2 | 0.7 |
| Leak Around Cannula Track | 1 | 0.4 |
| Pooling at Cannula Tip | 1 | 0.4 |
| Pooling of infusate into previous injection site (multiple infusions) | 1 | 0.4 |
| **Pain and Discomfort** | **78** | **27.4** |
| Headache | 57 | 20 |
| Pain (General) | 14 | 4.9 |
| Fatigue | 3 | 1.1 |
| Nausea and Vomiting | 2 | 0.7 |
| Neck Pain | 2 | 0.7 |
| **Systemic and Metabolic Complications** | **19** | **6.7** |
| Skin and Subcutaneous Tissue Disorders | 9 | 3.2 |
| Elevated Liver Enzymes | 2 | 0.7 |
| Elevated CK | 2 | 0.7 |
| Hematologic | 2 | 0.7 |
| Renal and Urinary Disorders | 2 | 0.7 |
| Hypertension | 1 | 0.4 |
| Rhabdomyolysis | 1 | 0.4 |

CL = contralateral, IL = ipsilateral, CN = cranial nerve, CSF = cerebrospinal fluid, CK = creatine kinase
